# Contact tracing in the hospital setting during the omicron wave of the coronavirus disease 2019 pandemic: persons and periods of concern for nosocomial infection prevention and control

**DOI:** 10.1017/ash.2024.80

**Published:** 2024-05-15

**Authors:** Lubna Sato, Yasuaki Tagashira, Narumi Shigeno, Yoshiaki Gu

**Affiliations:** 1 Department of Infectious Diseases, Tokyo Medical and Dental University (TMDU) Graduate School of Medical and Dental Sciences, Tokyo, Japan; 2 Department of Infection Prevention and Control, Tokyo Medical and Dental University (TMDU) Hospital, Tokyo, Japan; 3 TMDU Center for Infectious Disease Education and Analysis, Tokyo Medical and Dental University (TMDU), Tokyo, Japan

## Abstract

This study evaluating the efficacy of coronavirus disease 2019 contact tracing in the hospital setting during the omicron variant era found a high incidence of nosocomial severe acute respiratory coronavirus virus 2 (SARS-CoV-2) transmission in outbreaks, especially among individuals having close contact with infected persons. Identifying close contacts and outbreaks is essential to prevent nosocomial SARS-CoV-2 transmission.

## Introduction

Coronavirus disease 2019 (COVID-19) is an acute respiratory viral disease of pandemic proportions.^1^ With the development of treatments and vaccines and the emergence of viral variants, the severity of the disease and the associated mortality rate has decreased.^2^ At the outset of the pandemic, contact tracing extending retroactively 2 days prior to symptom onset was initially proposed for individuals who had had contact with persons with COVID-19.^3^ However, as the number of COVID-19 patients increased dramatically, contact tracing was discontinued, especially in the community setting.^4^

The inpatient wards of hospitals often house patients with multiple comorbidities and underlying conditions, including older age, malignancy, chronic lung disease, and use of corticosteroids or other immunosuppressive medications which are known to increase mortality in COVID-19.^5,6^ The introduction of severe acute respiratory coronavirus virus 2 (SARS-CoV-2) in such a setting can cause it to spread among healthcare workers (HCWs) and inpatients alike, potentially forming nosocomial outbreaks that eventually impact patient safety and hospital function.^7^ Merely relying on screening tests is insufficient to mitigate this threat.^8^ Contact tracing might serve as an effective risk assessment tool prior to implementing multifaceted, infection control measures. However, collecting contact information and the uncertainties inherent in the data pose challenges. A deeper consideration of the utility of contact tracing will inform ongoing discussions about measures for countering nosocomial transmission and serve as a reference for any future epidemic of respiratory diseases.

Herein, we retrospectively examined the real-world data on contact tracing at a university hospital in Tokyo during the omicron variant wave of the COVID-19 pandemic.

## Materials and methods

This retrospective cohort study was performed from January 2022 to March 2023 at Tokyo Medical and Dental University Hospital, an 813-bed, tertiary, referral hospital in central Tokyo containing 4 intensive care units, 16 general wards, and 2,457 HCWs. The general wards consist mainly of shared rooms, with the remaining rooms being single occupancy. Moreover, most of the intensive care units contain semi-separate rooms. When HCWs or inpatients in the hospital wards receive a diagnosis of COVID-19, an infection prevention and control (IPC) team collects detailed information about the individuals’ contact with other HCWs or inpatients to identify contacts up to 2 days prior to symptom onset. Exposure time, sharing a room, and personal protective equipment use were also considered when tracing contacts. A close contact was defined in accordance with the World Health Organization guidelines.^4^ Whenever multiple COVID-19 cases arose within 5 days with no clear epidemiological link, the IPC team considered these as forming an outbreak. Universal masking was strictly enforced in inpatients, and HCWs were required to wear eye shields and occasionally N95 respirators as part of the standard, precautionary infection prevention measures. Moreover, inpatients with symptoms were asked to receive tests proactively. Contact tracing was conducted in a similar manner. The institutional review board at Tokyo Medical and Dental University Hospital approved this study.

## Results

During the study period, contact tracing was performed in 781 COVID-19 cases. Among these, HCWs, patients, and others (administrative staff, students, visitors) accounted for 569 (72.8%), 195 (25.0%), and 17 (2.2%) cases, respectively, and 7,668 personnel had contact with COVID-19 patients in the hospital wards. Additionally, 782 (10.2%, 782 of 7,668) individuals were identified as close contacts. There were 229 (3.0%, 229 of 7,668) cases of epidemiologically defined, hospital-acquired COVID-19; 81 of these (10.4%, 81 of 782) originated in a close contact. Moreover, the incidence rate was higher in the outbreak setting than in the non-outbreak setting regardless of the presence of close contacts. Table 1 shows the detailed findings.

**Table 1.** Number of cases in each category, the outbreak setting and non-outbreak setting, and the total number

	Outbreak setting	Non-outbreak setting
Individuals exposed to persons with COVID-19, *n* = 7,668		
Persons with close contact, *n*, (%)	245 (31.3)	537 (68.7)
Persons without close contact, *n*, (%)	1,445 (21.0)	5,441 (79.0)
Individuals with close contact, *n* = 782		
Healthcare workers, *n*, (%)	75 (30.6)	211 (39.3)
Patients, *n*, (%)	167 (68.2)	313 (58.3)
Others*, *n*, (%)	3 (1.2)	13 (2.4)
COVID-19 cases thought to result from nosocomial transmission, *n* = 229		
Individuals with close contact, *n*, (incidence rate, %)	57 (23.3)	24 (4.5)
Individuals without close contact, *n*, (incidence rate, %)	96 (6.6)	52 (1.0)
COVID-19 patients who had close contact with infected individuals in the hospital resulting in nosocomial transmission (*n* = 81)		
Healthcare workers, *n*, (%)	15 (26.3)	11 (45.8)
Patients, *n*, (%)	42 (73.7)	12 (50.0)
Others*, *n*, (%)	0 (0)	1 (4.2)

Note. COVID-19, coronavirus disease 2019.

* Defined as nonmedical staff, medical students, student nurses, student radiology technicians, student pharmacists, and visitors.

**Figure 1.**
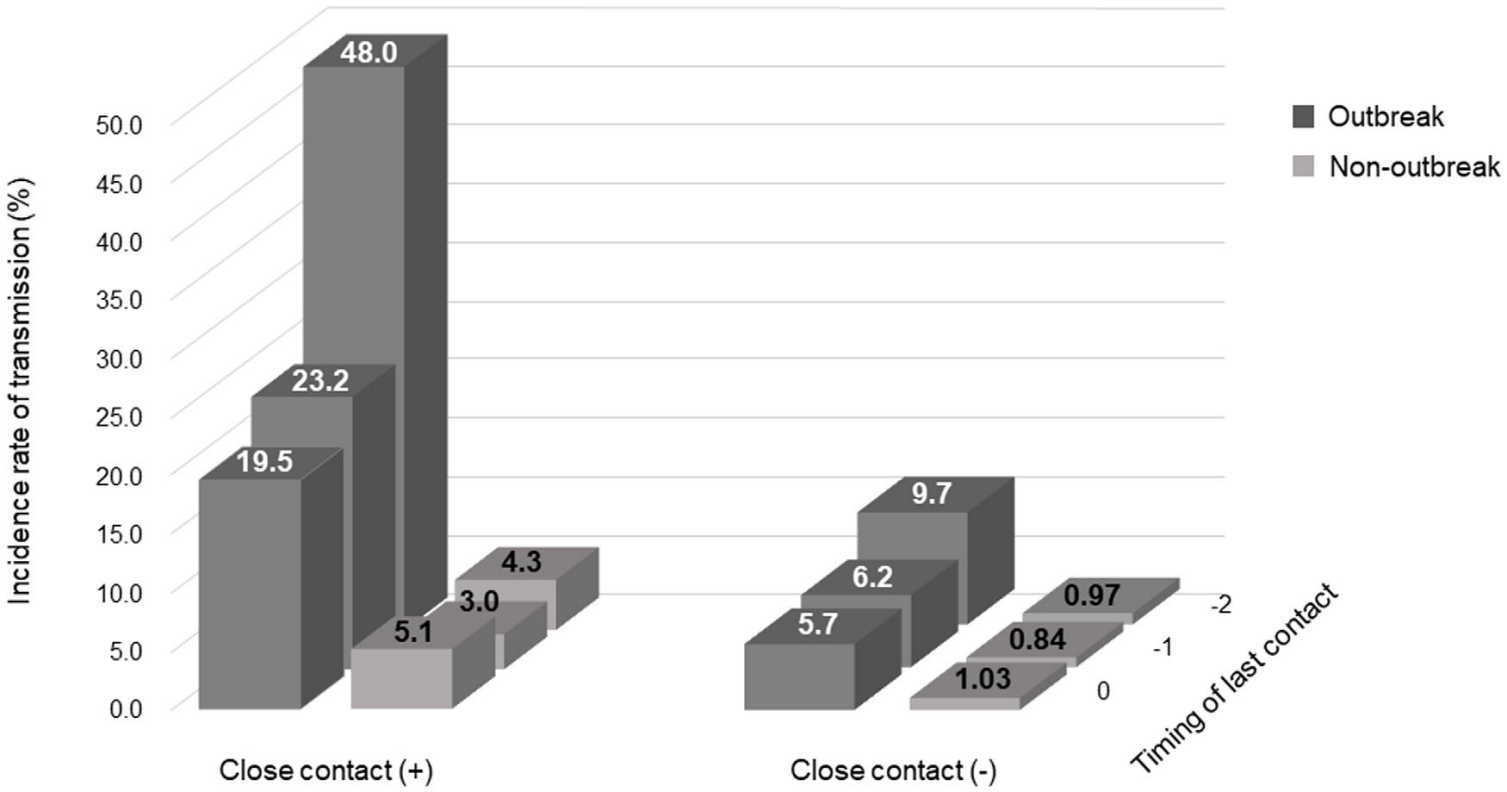
COVID-19 transmission rate by the time and presence of close contact with infected persons in the cluster and the non-cluster settings. Note: COVID-19, coronavirus disease 2019.

Figure 1 shows the incidence rate based on the time of the last contact before symptom onset, the presence of a close contact, and the outbreak setting. In the latter, the incidence rate was 19.5%–48.0% and 5.7%–9.7% in individuals with and without a close contact, respectively. In the non-outbreak setting, the incidence rate among individuals without a close contact was around 1.0%, while among those with a close contact, it ranged from 3.0% to 5.1%. The incidence rate did not differ significantly between the groups at 2 days before symptom onset.

## Discussion

The present study, which analyzed data on contact tracing in the nosocomial setting during the omicron wave of the COVID-19 pandemic, including the number of contacts and close contacts and the incidence rate, from various perspectives, found that the optimal strategy for infection prevention and control in this setting was rapid assessment for COVID-19 outbreaks in hospital wards, identification of close contacts of infected individuals, and isolation of patients with a high risk of infection.

The overall incidence rate among all contacts was only 3.0%, but among close contacts, it exceeded 10%, suggesting that identifying the latter is crucial for preventing and controlling the nosocomial spread of COVID-19. However, the study also found that the virus was transmissible before symptom onset. At present, there is no solution for this problem; effective, postexposure prophylaxis, such as for influenza, is desirable.

The present study has some limitations. It was retrospective and monocentric, thus limiting the generalizability of the findings. The contact lists were compiled on the basis of information provided by on-site HCWs, which may have decreased the accuracy of the data. As testing for asymptomatic carriers was not performed, the number of COVID-19 cases may be inaccurate. Because whole-genome sequencing was not conducted, precise transmission assessment was not possible. Moreover, the patients’ comorbidities and their impact on the mortality rate associated with nosocomial COVID-19 were not assessed. Further investigation would be warranted to determine the scope of contact tracing and its cost-effectiveness.

Identifying close contacts and outbreaks is crucial to preventing and controlling nosocomial COVID-19. Maintaining the vaccination rate and awareness of presenteeism among HCWs may also be important to this end. Verification using data from multiple facilities is necessary to construct a universal strategy in response to nosocomial COVID-19.
